# Native Mitral Valve Endocarditis Caused by* Neisseria elongata* subsp.* nitroreducens* in a Patient with Marfan Syndrome: First Case in Italy and Review of the Literature

**DOI:** 10.1155/2016/4956205

**Published:** 2016-10-10

**Authors:** Parrinello Rossella, Carità Patrizia, Triolo Fabio Oreste, Trapani Renato, Grassedonio Emanuele, Argano Vincenzo, Novo Giuseppina, Vallone Silvana, Fasciana Teresa, Verdecchia Massimo, Giammanco Anna, Midiri Massimo, Novo Salvatore

**Affiliations:** ^1^Department of Cardiology, University Hospital P. Giaccone, Palermo, Italy; ^2^Department of Cardiac Surgery, University Hospital P. Giaccone, Palermo, Italy; ^3^Department of Radiology, University Hospital P. Giaccone, Palermo, Italy; ^4^Department of Sciences for Health Promotion and Mother and Child Care “G. D'Alessandro”, University Hospital P. Giaccone, Palermo, Italy; ^5^Department of Radiology, University Hospital G. D'Annunzio, Chieti, Italy

## Abstract

*Neisseria elongata* (NE) is an aerobic Gram-negative organism that constitutes part of the commensal human normal oropharyngeal flora. Although previously considered not to be pathogenic, it has been recognized as an occasional cause of significant infections in humans. We report here the first case in Italy of infective endocarditis of a native prolapsing mitral valve in a patient with Marfan syndrome, caused by NE subspecies* nitroreducens* which has been rarely isolated from clinical specimens. The culprit organism has been confirmed by mass spectrometry directly from the positive blood culture, as previously reported. The amplified gene has been deposited in GenBank under accession number KT591873. In spite of the reported aggressive nature of NE, clinical remission was promptly obtained, there being no requirement for surgery.

## 1. Introduction

*Neisseria elongata* (NE) is an aerobic Gram-negative organism that constitutes part of the commensal human normal oropharyngeal flora [1]. Several biochemical characteristics can be used to differentiate the organism into three subspecies:* elongata*,* glycolytica*, and* nitroreducens*. Although previously considered not to be pathogenic, it has been recognized as an occasional cause of significant infections in humans, such as infective endocarditis (IE), septicaemia, and osteomyelitis. The subspecies* nitroreducens* is the most frequently involved [1–4]. We report here the first case in Italy of IE of a native prolapsing mitral valve caused by NE subsp.* nitroreducens* in a patient with Marfan syndrome.

## 2. Case Report

A 40-year-old Italian man with Marfan syndrome was admitted to our hospital because of remittent fever reaching up to 39, lasting for one week. Therapy with oral amoxicillin-clavulanic acid, prescribed by his general physician six days earlier, was ineffective in improving his symptoms. He denied any recent dental procedures or odontogenic infections, nor invasive procedures.

In 1992, at the age of 17, he underwent composite aortic valve and root replacement with a composite graft that consisted of Starr Edward's aortic prosthesis and a suitably sized Dacron tube. A routine transthoracic echocardiogram (TTE) performed one year before hospital admission showed mitral valve prolapse with severe mitral regurgitation.

On admission the following parameters were detected: arterial blood pressure of 120/60 mmHg, heart rate of 117 bpm (regular rhythm), body temperature of 38°C, and arterial oxygen saturation of 97%. The physical examination revealed a systolic murmur at the left sternal border. Neither mucocutaneous signs related to infective endocarditis, such as Janeway lesions, splinter haemorrhages, Osler nodes, and conjunctival petechiae, nor active intraoral lesions were detected; the neurological examination was unremarkable.

**Figure 1:**
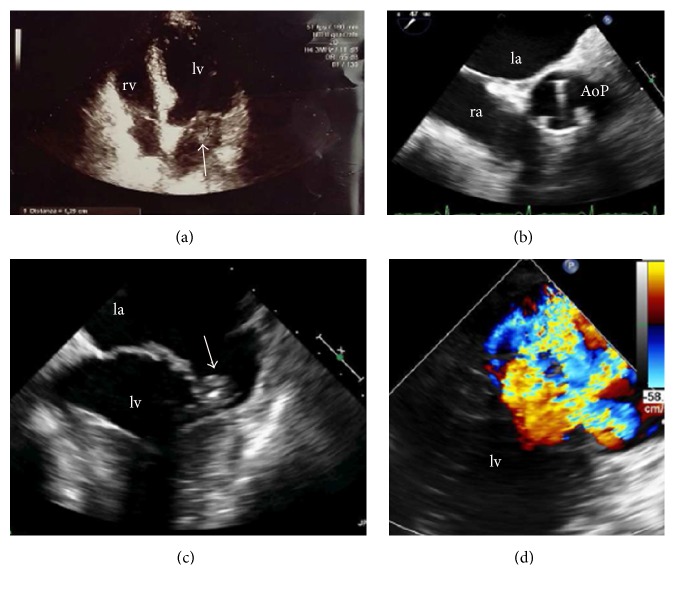
TTE 4-chamber views showing (a) a vegetation arising from the posterior leaflet of mitral valve. The vegetation was confirmed with TOE (c) that also documented a severe mitral regurgitation (d) and the prosthetic aortic valve (b). la: left atrium, lv: left ventricle, ra: right atrium, rv: right ventricle, and AoP: prosthetic aortic valve.

**Figure 2:**
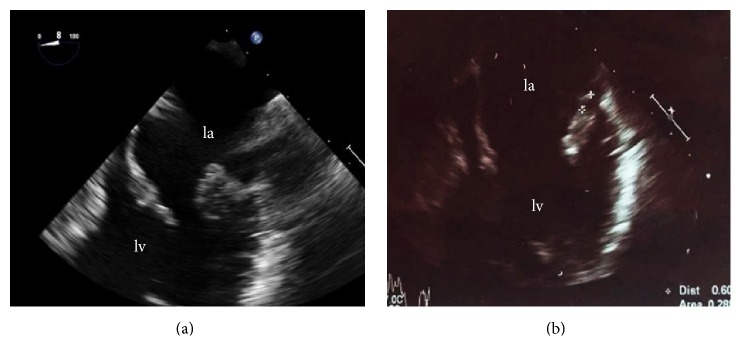
TOE 4 chambers: the admission of TOE on (a) shows a vegetation on the posterior leaflet of mitral valve. The follow-up TOE (b) confirmed the persisting of the vegetation of reduced dimensions, compared with the previous exam (a), despite clinical remission.

Blood test demonstrated normal white blood cell count but elevated levels of C-reactive protein (>75 mg/L). The electrocardiogram showed sinus tachycardia and left ventricular hypertrophy. Transthoracic and transesophageal echocardiography (TOE) showed left atrium enlargement and left ventricular hypertrophy with normal LV ejection fraction; it also revealed the presence of a vegetation on the posterior leaflet of mitral valve (12 mm in diameter) (Figure 1) and confirmed the severity of mitral regurgitation. The patient underwent a computed tomography (chest, abdomen, and brain) that excluded signs of infection or haematogenous dissemination. As suggested by the current European guidelines, an empirical antimicrobial therapy for native valve endocarditis was started with intravenous amoxicillin/clavulanic acid (875 mg/125 mg × 3 die) and gentamicin (80 mg × 2 die). On hospital day 3 the patient became apyretic and he remained so throughout the entire clinical course; C-reactive protein progressively declined. After seven days, the admission blood cultures grew Gram-negative coccobacilli, susceptible to a broad spectrum of antibiotics, including gentamicin and ceftazidime. Furthermore, through a molecular approach based on 16 S ribosomal DNA sequencing, the isolate was identified to be* N. elongata*. The patient was switched from amoxicillin to intravenous ceftazidime (2 g × 3 die) in addition to gentamicin. After two weeks of appropriate treatment C-reactive protein returned to normal. After 18 days the blood cultures obtained during the patient's antibiotic therapy were negative. However, despite clinical and laboratory remission, TOE performed on day 19 detected two little vegetations (maximum diameter 6 mm) on the mitral valve (Figure 2). The patient was discharged on day 21, in good clinical condition with the recommendation to continue the antibiotic therapy (intramuscular gentamicin and intramuscular ceftazidime) for other three weeks. A follow-up TTE performed one month after hospital discharge showed the complete resolution of the vegetation.

## 3. Discussion

We report here the first case in Italy of infective endocarditis caused by NE subsp.* nitroreducens*, which has been rarely isolated from clinical specimens. To the best of our knowledge, more than 20 cases of NE endocarditis have appeared in the literature, of which 18, including the present case, fulfilled the modified Dukes criteria for a definite diagnosis of infective endocarditis [5]. Preexisting heart diseases (valvular or congenital heart disease) are considered as risk factors for* N. elongata* endocarditis [6–8]. However, some cases have also been occasionally reported in patients without previous apparent cardiac disease [9].

*Neisseria*-induced endocarditis seems to have a poor prognosis and the disease course might be complicated and potentially fatal. A review of definitive or possible cases of* N. elongata* endocarditis showed that approximately half of the patients underwent surgical treatment [10]. Moreover, it often results in severe cardiac and systemic complications such as systemic embolization, heart failure, and myocardial abscess [11, 12]. Thus, its involvement in an infective process should be promptly treated and managed with close follow-up. Some authors have suggested that clinicians should consider extended antibiotic treatment and early surgical evaluation [13]. To date, the drug susceptibility testing of NE has not yet been standardized. However, this organism is generally considered to be susceptible to a wide range of antibiotics [14]. Many cases of endocarditis are treated with beta-lactams, often in combination with aminoglycosides, for four to six weeks [10]. However, appropriate protocols for antibacterial treatment should be established with further cases.

In conclusion, to the best of our knowledge, we present the first case in Italy of definite diagnosis of IE caused by NE subspecies* nitroreducens*. The patient underwent a previous replacement of aortic valve, but the infection involved the native prolapsing mitral valve. No recent invasive procedures or oral manipulation was reported as major risk factors. The last time the patient went to the dentist was several years before the hospital admission. Hence, we speculate that NE entered the bloodstream during unrecognized oral events, such as daily dental care. Cases originating from a skin infection have also been reported [15]. The culprit organism has been confirmed by mass spectrometry directly from the positive blood culture, as previously reported. The amplified production including 1416 nucleotides of the 16SrRNA gene of NE subsp.* nitroreducens* was deposited in GenBank under accession number KT591873, as previously described [16]. The rapid identification has enabled us to administer a correct chemical therapy. In spite of the reported aggressive nature of NE, clinical remission was promptly obtained with intravenous ceftazidime in addition to gentamicin, there being no requirement for surgery during the acute phase of the disease.

No local or systemic complication was reported.
